# Anti-Inflammatory Flavonoids from *Agrimonia pilosa* Ledeb: Focusing on Activity-Guided Isolation

**DOI:** 10.3390/molecules29020283

**Published:** 2024-01-05

**Authors:** Mijin Park, Dahye Ryu, Jwayeong Cho, Kang-Mo Ku, Young-Hwa Kang

**Affiliations:** 1 Department of Horticultural Sciences, College of Agriculture & Life Sciences, Kyungpook National University, Daegu 41566, Republic of Korea; mj-7311@hanmail.net (M.P.); dahyeryu0507@hanmail.net (D.R.); chocho7023@kist.re.kr (J.C.); 2 Department of Plant Biotechnology, Korea University, Seoul 02841, Republic of Korea; ku_km@korea.ac.kr

**Keywords:** *Agrimonia pilosa*, anti-inflammatory compounds, bioactivity-guided isolation, nitric oxide inhibitory activity

## Abstract

To elucidate the anti-inflammatory properties and constituents of *Agrimonia pilosa* Ledeb. (*A. pilosa*), a comprehensive investigation was conducted employing activity-guided isolation. The anti-inflammatory effects were evaluated through an in vitro nitric oxide (NO) assay on lipopolysaccharide (LPS)-treated RAW 264.7 macrophage cells. Seven bio-active compounds with anti-inflammatory properties were successfully isolated from the butanol fraction and identified as follows: quercetin-7-*O*-β-d-rhamnoside (**1**), apigenin-7-*O*-β-d-glucopyranoside (**2**), kaempferol-7-*O*-β-d-glucopyranoside (**3**), quercetin (**4**), kaempferol (**5**), apigenin (**6**), and apigenin-7-*O*-β-d-glucuronide-6″-butylester (**7**). All isolated compounds showed strong NO inhibitory activity with IC_50_ values ranging from 1.4 to 31 µM. Compound **6** demonstrated the most potent NO inhibition. Compound **7**, a rare flavonoid, was discerned as a novel anti-inflammatory agent, ascertained through its inaugural demonstration of nitric oxide inhibition. Subsequently, a comprehensive structure-activity relationship (SAR) analysis was conducted employing eight flavonoids derived from *A. pilosa*. The outcomes elucidated that flavones exhibit superior NO inhibitory effects compared to flavonols, and the aglycone form manifests greater potency in NO inhibition than the glycone counterpart. These results highlight *A. pilosa* as a promising source of effective anti-inflammatory agents and indicate its potential as a health-beneficial dietary supplement and therapeutic material.

## 1. Introduction

*Agrimonia pilosa* (*A. pilosa*), also known as hairy agrimony, is an herbaceous flowering plant belonging to the family Rosaceae, order Rosales. The aerial parts of *A. pilosa* have been used in traditional medicine in Korea, Japan, and China [1], and the plant is native to East Asia [2]. *A. pilosa* contains diverse chemical components such as flavonoids [3], isocoumarins [4,5], tannins [6], and terpenoids [7]. Recent studies reported that *A. pilosa* extracts demonstrated broad physiological activities, such as antioxidant, anti-inflammatory, anti-cancer, nitric oxide scavenging, and α-glucosidase inhibitory activities [8,9,10,11,12]. In a previous study, five phenolic compounds, namely aromadendrin, dihydrokaempferol 3-*O*-β-d-glucoside, quercitrin, aglimonolide-6-*O*-α-d-glucoside, and loliolide, were found to be NO scavengers in this plant [12]. In addition, the ethanol fraction from *A. pilosa* extract showed excellent anti-inflammatory activity, including NO scavenging, inflammatory cytokine suppression, and inflammatory enzyme repression activity [13]. As part of our comprehensive investigation of anti-inflammatory compounds from *A. pilosa*, the constituents of *A. pilosa* were investigated. The macrophage-derived NO that is present under treatment with cytotoxic molecules such as LPS, is considered to play a key role in inflammation and immune response [14]. Thus, the inhibition of NO production can be used as a criterion for the evaluation of anti-inflammatory effects. The aim of this study is to investigate the anti-inflammatory compounds present in the butanol fraction of *A. pilosa* (APB) that exhibit the most potent anti-inflammatory effects and to elucidate their chemical structures using various spectroscopic techniques such as UV spectroscopy, mass spectrometry (MS), and one-dimensional (1D) and two-dimensional (2D) nuclear magnetic resonance (NMR) spectroscopy. Furthermore, this study aims to evaluate and compare the anti-inflammatory activities of these compounds by measuring their inhibitory impact on nitric oxide (NO) production in lipopolysaccharide (LPS)-induced RAW 264.7 macrophages, thereby providing valuable insights into the potential therapeutic applications of *A. pilosa* and their active compounds in conditions associated with inflammation and immune response.

## 2. Results and Discussion

### 2.1. Activity-Guided Isolation

In our preliminary screening, the methanol extract from *A. pilosa* exhibited excellent anti-inflammatory (AIN) activities. While previous studies have reported the anti-inflammatory activities of agrimonolide and tiliroside derived from *A. pilosa* [12,13], an exhaustive investigation on anti-inflammatory activity-guided isolation is currently lacking in the literature. Consequently, to elucidate the anti-inflammatory principle underlying *A. pilosa*, an anti-inflammatory activity-guided isolation was performed.

**Figure 1:**
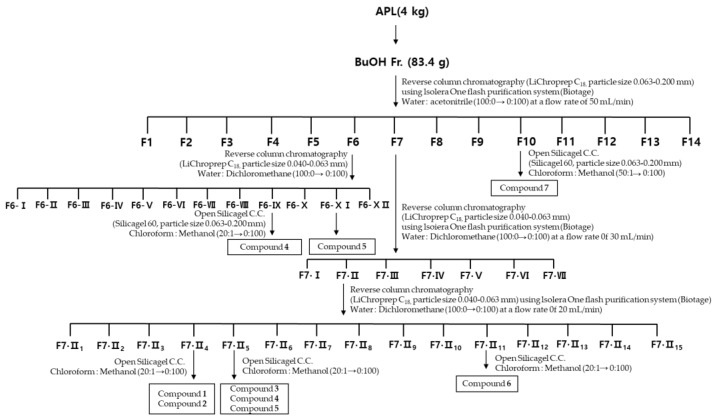
Activity-guided isolation of seven compounds from the butanol fraction of *A. pilosa*.

Anti-inflammatory activity was evaluated by nitric oxide production in lipopolysaccharide (LPS)-treated RAW 264.7 macrophage. In order to elucidate the anti-inflammatory compounds of *A. pilosa*, the methanol extract of AP (APM) was obtained and fractionated with hexane, ethylacetate, and butanol, successively. At non-cytotoxic concentrations (0–40 µg/mL) with or without the presence of 1 µg/mL LPS, the inhibitory effects of the *A. pilosa* extract and solvent fractions on macrophage activation were investigated. The butanol fraction of *A. pilosa* (APB) showed the strongest inhibitory effect on LPS-induced NO production. The anti-inflammatory activity of APB was further studied. The APB (83.4 g), the highest active fraction, was subjected to C_18_ column chromatography (CC) to yield fourteen fractions (Figure 1). The anti-inflammatory activities of fourteen fractions, F1~F14, were measured through nitric oxide (NO) production in lipopolysaccharide (LPS)-stimulated RAW 264.7 macrophages (Table 1). Among fourteen fractions, F6, F7, and F10 showed strong NO inhibitory effects. Three active fractions from the butanol fraction of *A. pilosa* (APB) were fractionated with various separation techniques, successively. Fraction F6 was subjected to C_18_ column chromatography (CC), and twelve fractions were obtained from F6. Active fractions, F6-IX (7.4 g) and F6-XI (5.1 g) from F6 were subjected to silica gel CC to yield compounds 4 (74.1 mg) and 5 (54.0 mg), respectively. Fraction F7 was subjected to C_18_ CC to yield seven fractions, F7-I~F7-VII. Among the fractions from F7, F7-II, the most active fraction, was rechromatographed using C_18_ CC to yield fifteen fractions, F7-II_1_~F7-II_15_. Compounds **1** and **2** were derived from F7-II_4_, while compounds **3**, **4** (15.2 mg), and **5** (13.2 mg) originated from F7-II_5_, using silica gel CC. Compounds **4** and **5** were isolated from F6 and F7. Additionally, compound **6** was successfully isolated from F7-II_11_ using silica gel CC. Compound **7** was obtained from Fraction F10 subjected to silica gel column chromatography. Subsequently, quantitatively, the isolated amounts of anti-inflammatory compounds from these highly active fractions were determined as follows: compound **1** (23 mg), compound **2** (8 mg), compound **3** (197 mg), compound **4** (88 mg), compound **5** (67 mg), compound **6** (8 mg), and compound **7** (28 mg). This precise quantification provides quantitative insights into the distribution of these bioactive entities within the most potent fraction, thereby establishing a foundation for further detailed analyses of their therapeutic potential and mechanisms of action. Compounds **3**, **4**, and **5** were the primary anti-inflammatory compounds within the butanol fraction of *A. pilosa*.

### 2.2. Identification of Bio-Active Compounds

**Figure 2:**
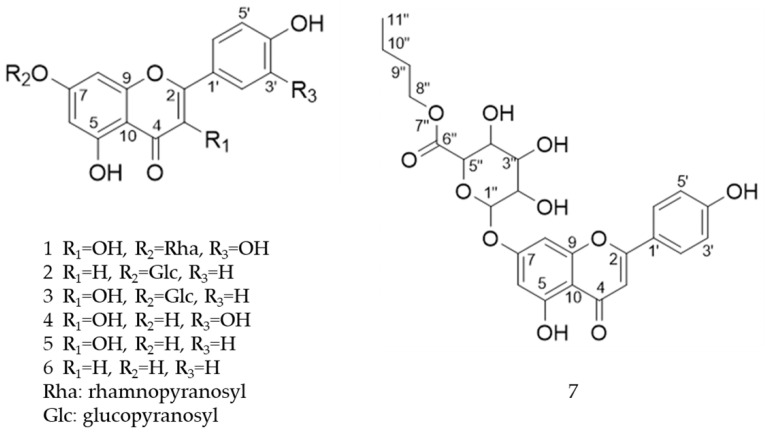
The structures of compounds **1**–**7** from *A. pilosa*.

Following a rigorous bioactivity-guided isolation strategy, seven distinct anti-inflammatory compounds were successfully isolated (Figure 2). The purity of all isolated compounds was determined by HPLC analysis. When the purity of compounds **1**–**7** was greater than 95%, structural characteristic analysis was performed. In order to identify the structural characteristics, the set of spectroscopic data, including retention times of HPLC profiles, mass spectral data, and NMR chromatograms, was estimated. The structure of the compounds was also confirmed using comprehensive spectroscopic analysis data by comparison of the literature [15,16,17,18,19,20,21,22,23,24] and their 1H-NMR and MS data. Their analytical data were as follows.

Quercetin-7-O-β-*d-rhamnoside* (**1**). Yellow, amorphous powder; HPLC TR 18.1 min (purity > 94%); UV (MeOH) λmax (log ε) 253 (2.40), 354 (2.55) nm; 1H NMR (MeOH-d4, 500 MHz) (Table S1); ESI-MS positive *m*/*z*, 449 [M + H]^+^ (Calculated for C_21_H_20_O_11_, 448).
*Apigenin-7-O-β-d-glucopyranoside* (**2**). Pale yellow; amorphous powder; HPLC TR 18.1 min (purity > 94%); UV (MeOH) λmax (log ε) 241 (2.38), 269 (2.43), 311 (2.45) nm; 1H NMR (DMSO-d6, 500 MHz) (Table S1); ESI-MS positive *m*/*z*, 433 [M + H]^+^ (Calculated for C_21_H_20_O_10_, 432).
*Kaempferol-7-O-β-d-glucopyranoside* (**3**). Yellow; amorphous powder; HPLC TR 18.8 min (purity > 97%); UV (MeOH) λmax (log ε) 227 (2.35), 270 (2.43), 335 (2.52) nm; 1H NMR (DMSO-d6, 500 MHz) (Table S1); ESI-MS positive *m*/*z*, 449 [M + H]^+^ (Calculated for C_21_H_20_O_11_, 448).
*Quercetin* (**4**). Yellow; amorphous powder; HPLC TR 20.7 min (purity > 97%); UV (MeOH) λmax nm (log ε) 253 (2.40), 354 (2.55); 1H NMR (MeOH-d4, 500 MHz) (Table S1); ESI-MS positive *m*/*z*, 303 [M + H]^+^ (Calculated for C_15_H_10_O_7_, 302).
*Kaempferol* (**5**). Yellow; amorphous powder; HPLC TR 20.9 min (purity > 96%); UV (MeOH) λmax (log ε) 253 (2.40), 354 (2.55) nm; 1H NMR (DMSO-d6, 500 MHz), (Table S1); ESI-MS positive *m*/*z*, 287 [M + H] ^+^ (Calculated for C_15_H_10_O_6_, 286).
*Apigenin* (**6**). Yellow; amorphous powder; HPLC TR 22.4 min (purity > 98%); UV (MeOH) λmax (log ε) 253 (2.40), 276 (2.44), 296 (2.47) nm; 1H NMR (DMSO-d6, 500 MHz), (Table S1); ESI-MS positive *m*/*z*, 271 [M + H]^+^ (Calculated for C_15_H_10_O_5_, 270).
*Apigenin-7-O-β-d-glucuronide-6″-butylester* (**7**). Pale pink, amorphous powder; HPLC TR 19.9 min (purity > 91%); mp 259–264 °C; [α]^25^_D_ −25.08° (c 0.1, MeOH); UV (MeOH) λmax (log ε) 267 (2.43), 337 (2.53) nm; 1H and 13C NMR (DMSO-d6, 500/125 MHz), HMBC, COSY, DEPT and HSQC; HR-ESI-MS positive *m*/*z*, 503.1557 [M + H]^+^ (Calculated for C_25_H_26_O_11_, 502.1633), (Table 2 and Figures S1–S6).

**Table 2:** NMR Spectroscopic Data of Compound **7**.

Position	*δ_C_*, Type	*δ*_H_ (mult, *J*, int)	HMBC	COSY
**2**	165.66, C			
**3**	102.83, CH	6.69 (s, 1H)	**4, 5, 8, 10, 1′**	
**4**	182.93, C			
**5**	161.81, C			
**6**	99.75, CH	6.51 (d, *J* = 2.2 Hz, 1H)	**5, 8, 10**	**8**
**7**	163.21, C			
**8**	94.65, CH	6.82 (d, *J* = 2.2 Hz, 1H)	**5, 6, 9, 10**	**6**
**9**	157.77, C			
**10**	106.05, C			
**1′**	121.94, C			
**2′**	128.24, CH	7.90 (d, *J* = 8.9 Hz, 2H)	**2, 5, 6′**	**6′**
**3′**	115.67, CH	6.97 (d, *J* = 8.9 Hz, 2H)	**1, 5, 8, 10, 5′**	
**5′**	115.67, CH	6.97 (d, *J* = 8.9 Hz, 2H)	**1, 5, 8, 10, 3′**	
**6′**	128.24, CH	7.90 (d, *J* = 8.9 Hz, 2H)	**2, 5, 2′**	**2′**
**1″**	100.10, CH	5.21 (d, *J* = 7.6 Hz, 1H)	**7**	**3″**
**2″**	73.06, CH	3.26 (dd, *J* = 9.5, 11.6 Hz, 1H)		
**3″**	75.71, CH	3.54 (dd, *J* = 9.5, 11.6 Hz, 1H)	**1″, 2″, 3″, 4″, 5″**	**1″**
**4″**	71.29, CH	3.70 (m, 1H)	**3″,** **5″**	**5″**
**5″**	75.37, CH	4.17 (d, *J* = 9.5 Hz, 2H)	**1″, 3″, 4″**	**3′′, 4′′**
**6″**	169.15, C			
**8″**	65.04, CH_2_	4.23 (m, 2H)	**9″, 10″**	**9′′**
**9″**	30.26, CH_2_	1.68 (m, 2H)	**8″, 10″, 11″**	**8″, 10″**
**10″**	18.67, CH_2_	1.42 (m, 2H)	**8″, 9″, 11″**	**9″, 11″**
**11″**	12.56, CH_3_	0.91 (t, *J* = 7.4 Hz, 3H)	**9″, 10″**	**10″**

Recorded in DMSO-*d*_6_ at 500/125 MHz (TMS as internal standard); chemical shifts, multiplicity, and coupling constants (*J*, Hz) were assigned by means of ^1^H, ^13^C NMR, and 2D-NMR data.

Seven compounds, based on these analytical data, were identical as quercetin-7-*O*-β-d-rhamnoside (**1**), apigenin-7-*O*-β-d-glucopyranoside (**2**), kaempferol-7-*O*-β-d-glucopyranoside (**3**), quercetin (**4**), kaempferol (**5**), apigenin (**6**), and apigenin-7-*O*-β-d-glucuronide-6″-butylester (**7**), respectively. Their chemical structures are depicted in Figure 2. To identify the structure of compound **7**, various NMR techniques, including HMBC, COSY (Table 2), DEPT, and HSQC (Figures S4 and S5), were used, and the molecular weight of compound **7** was measured by HR-ESI-MS analysis. The molecular formula of compound **7** was determined to be C_25_H_26_O_11_ by HR-ESI-MS data (*m*/*z* 503.1557 [M + H]**^+^**). The 1H and 13C NMR spectra of compound **7** were similar to those of apigenin-7-*O*-β-d-glucopyranoside. The 1H NMR spectrum of compound **7** has resonances assignable to two meta coupled aromatic protons at δH 6.51 (H-6) and 6.82 (H-8), four A2X2 type aromatic ring protons at δH 6.97 (H-3′, 5′) and 7.90 (H-2′, 6′), and one-proton singlets at δH 6.69 (H-3).

The 13C NMR and DEPT NMR spectrum exhibited resonances assignable to apigenin (Api), one carbonyl at δC 182.93 (C-4), six methane carbons at δC 102.83 (C-3), 94.65 (C-8), 128.24 (C-2′, 6′), and 115.67 (C-3′, 5′), six oxygen-bearing carbons at δC 165.66 (C-2), 161.81 (C-5), 163.21 (C-7), 157.77 (C-9), and three quaternary carbons at δC 157.77 (C-9), 106.05 (C-10), and 121.94 (C-1′) [18]. We also identified β-d-glucuronide by comparing its spectral data with literature values [19] along with a β-d-glucuronide group with anomeric protons at δH 5.21 (H-1″) [20]. Furthermore, the 1H NMR spectrum of compound **7** showed three methylene with three multiplet signals at δH 4.23 (H-8″), δH 1.68 (H-9″) and δH 1.42 (H-10″), and one methyl with triplet signals at 0.91 (H-11″). These data were assigned as the butyl moiety (CH_2_-CH_2_-CH_2_-CH_3_) in the structure of compound **7**. The butyl moiety was also confirmed by DEPT spectral data. The DEPT data showed the peaks of -CH_2_- (18.67, 30.26, and 65.04 ppm) and -CH_3_ (12.56 ppm) in the butyl chain. Based on the analysis of spectroscopic data, the structure of compound **7** was determined to be apigenin-7-*O*-β-d-glucuronide-6″-butylester.

### 2.3. Anti-Inflammatory Effects of Isolated Compounds

Nitric oxide (NO) serves as a signaling molecule pivotal in the pathogenesis of inflammation. Under normal physiological conditions, it exerts an anti-inflammatory effect. Conversely, in abnormal situations characterized by overproduction, NO is regarded as a pro-inflammatory mediator that instigates inflammation [13]. Macrophage-derived nitric oxide (NO) generated during treatment with cytotoxic molecules like LPS is recognized to play an important role in inflammation and immune responses [14]. Consequently, the inhibition of NO production stands as a valuable criterion for assessing anti-inflammatory effects. Compounds **1**–**7** were tested for anti-inflammatory activity through NO inhibition in LPS-treated RAW 264.7 macrophages at non-cytotoxic concentration ranges (0–20 μM). When the isolated compounds were treated in the NO production system, NO accumulation was significantly inhibited in a dose-dependent manner. The highest NO inhibitory ability was obtained from compound **6** (apigenin), with an IC_50_ value of 1.43 μM, followed by compounds **5** > **2** > **7** > **4** > **3** > **1** (IC_50_ values of 5.75, 8.03, 14.68, 19.51, 22.24, and 31.26 μM, respectively (Table 3)).

Eighteen anti-inflammatory compounds including ten flavonoids, five terpenoids, and three isocoumarins from *A. pilosa* were reported [25]. Their nitric oxide (NO) inhibitory effects demonstrated a range of 38 to 177 μM for flavonoids, 125–132 μM for terpenoids, and 25–76 μM for isocoumarins. Within the pool of 58 flavonoids isolated from *A. pilosa*, ten specific flavonoids, including tiliroside, catechin, pilosanidin A, B, pilosanol A, B, C, N, naringin, and dihydrokaempferol glucopyranoside, were reported as anti-inflammatory compounds, exerting their effects through NO inhibition. Among the known anti-inflammatory compounds in *A. pilosa*, pilosanidin A demonstrated the highest NO inhibitory ability with an IC_50_ value of 38 μM [25]. Notably, the seven flavons and flavonols isolated in this study exhibited superior anti-inflammatory activity compared to pilosanidin A (38 μM), underscoring the efficacy of the activity-guided isolation methodology in identifying potent bioactive compounds. Seven compounds isolated in this study were first reported as NO inhibitors in this plant. Compound **7**, identified as a rare flavonoid, emerged as a novel anti-inflammatory agent, as evidenced by its inaugural demonstration of nitric oxide inhibition. The study highlighted the significance of this compound, given its rarity and substantial anti-inflammatory properties. Additionally, it is worth mentioning that the methyl ester of apigenin-7-*O*-β-d-glucuronide, previously isolated from the ethyl acetate leaf extract of Manilkara zapota, exhibited noteworthy anti-cancer efficacy against breast cancer.

This comprehensive research not only sheds light on the anti-inflammatory potential of specific compounds in *A. pilosa* but also suggests avenues for further exploration. The in-depth investigation of compound **7** is deemed indispensable, as it not only serves as a foundational element but also lays the groundwork for unraveling the diverse spectrum of biological activities associated with this botanical entity.

### 2.4. Structure-Activity Relationship of Flavones and Flavonols from A. pilosa

Eight compounds from A. pilosa were selected for a structure–activity relationship study. The structural determinants influencing the inhibitory activity of flavonoid aglycone and its glucosylated derivatives from *Agrimonia pilosa* on nitric oxide (NO) were systematically investigated and summarized in Table 4. This investigation aimed to elucidate the intricate connections between the structural characteristics of flavonoids and their anti-inflammatory properties, providing a nuanced understanding of the molecular determinants governing the observed pharmacological effects. The presence of a hydroxyl group at position 3′ on the B ring of the flavone moiety influenced NO inhibitory activity, with a decrease observed as follows: apigenin (IC_50_ = 3.69 μM) demonstrating greater efficacy compared to luteolin (IC_50_ = 4.62 μM). Additionally, the hydroxyl group at position 3 on the C ring emerged as a critical factor for NO inhibitory activity, with the flavonol moiety featuring a hydroxyl group at this position leading to a diminished NO inhibitory effect. The descending order of NO inhibitory activity was observed as follows: apigenin (IC_50_ = 3.69 μM) > luteolin (IC_50_ = 4.62 μM) > kaempferol (IC_50_ = 14.43 μM) > quercetin (IC_50_ = 19.50 ± 1.71 μM). The examination of NO inhibitory activities of flavones’ and flavonols’ glycosides revealed a decreasing trend according to the order of Apigenin-Glc (IC_50_ = 8.03 μM) > Luteoline-Glc (IC_50_ = 14.37 μM) > Kaempferol-Glc (IC_50_ = 22.24 μM) > Quercetin-Glc (IC_50_ = 31.27 μM). Increased –OH groups and glycosylation correlated with diminished NO inhibitory effects, suggesting that the number of –OH groups and glycosylation of flavones are crucial factors in NO inhibitory activity. The effects of hydroxyl group and glycosylation on activity were reported in a study comparing the flavone and flavone-Glc structure and antioxidant activity [26]. The bioactivity–structure relationship for the antioxidant activity of flavonoids from *A. pilosa* showed that the glycosidation of flavonoids at C-6 in the A-ring enhanced the stability of free radicals, thereby increasing antioxidant capacity [27]. Meanwhile, glycosidation at C-7 in the A-ring and the hydroxyl group at C-3 in the C-ring significantly decreased NO inhibitory activity in the present study.

## 3. Materials and Methods

### 3.1. General Experimental Procedure

The UV spectra were obtained from UV spectrometer (Power Wave XS, Bio-Tek Instrument, Winooski, VT, USA). Thin-layer chromatography (TLC) was carried out on precoated Merck silica gel 60 F_254_ and RP-C_18_ F_254_ plates from Merck (Darmstadt, Germany). Silica gel (63–200 µm particle size) and RP-C_18_ (40–63 µm particle size) for column chromatography were from Merck (Darmstadt, Germany). Isolera One flash purification system (Biotage, Sweden), equipped with a reverse phage preparation column (340 g, LiChroprep C18, particle size 40–63 µm, Merck), was used for effective separation. HPLC analysis for purification assignment was performed on a Shimazu LC20A equipped with a DAD detector (set at 280 nm and 360 nm) and a YMC-Triart C18 column (4.6 × 250 mm, 5 μm; YMC, Japan) held at 40 °C (CTO-20A, Shimadzu, Kyoto, Japan), under the following solvent program: solvent A, 0.1% H_3_PO_4_ in water and solvent B, 0.1% H_3_PO_4_ in methanol, starting from 20% B to 30% B in 5 min, to 50% B in 10 min, to 70% B in 15 min, to 100% B in 20 min, and to 20% B in 35 min. Flow rate was set at 1 mL/min. NMR spectra were acquired on an Avance III 500 spectrometer. Spectra were recorded in MeOH-d4 and DMSO-d6 using Bruker with tetramethylsilane as the internal standard. The reagents including 3-[4,5dimethylthiazol-2-yl]-2,5-diphenyltetrazolium bromide (MTT), dimethyl sulfoxide (DMSO), naphthylethylenediamine dihydrochloride, and sulfanilamide were purchased from Sigma Aldrich Ltd. (St. Louis, MO, USA). Luteolin, luteolin-7-glucopyronoside, and qercetin-7-glucopyranoside were obtained from *A. pilosa* in our previous study. HPLC solvents were purchased from Thermo Fisher Scientific Korea Ltd. (Seoul, Republic of Korea). All other chemicals were analytical grade.

### 3.2. Plant Materials

Dried aerial parts of *Agrimonia pilosa* (*A. pilosa*) were procured from Yangnyeong market in Daegu, Korea, with a verified origin in Korea. Prof. Kang S.H., affiliated with the Department of Natural Medicine Resources at Semyung University in Jecheon, Republic of Korea, conducted the botanical identification. A voucher specimen (NP-116) was deposited at the herbarium of Kyungpook National University (Daegu, Republic of Korea). Plant material was chopped and stored at room temperature until the commencement of experiments.

### 3.3. Exraction and Isolation

Dried *A. pilosa* plant (4 kg) was extracted with MeOH (9 × 10 L, at 65 °C, 4 h each). The extract was filtrated through Whatman no. 2 paper and then evaporated in a vacuum at 70 °C using a rotary evaporator (JP/N 1000S-W; Eyela, Tokyo, Japan) and water bath (Digital Water Bath SB-651; Rikakikai, Tokyo, Japan) to remove the solvent, yielding a methanol extract (440 g). The methanol extract was suspended in distilled water (10 L) and extracted successively with hexane (15 × 2 L), ethyl acetate (15 × 2 L), and n-butanol (15 × 2 L). The hexane, ethyl acetate, and butanol fractions were concentrated at 70 °C and produced hexane (96.0 g), ethyl acetate (165.0 g), and butanol (83.4 g) residues, respectively. The butanol fraction (APB), the highest active fraction., was separated using an Isolera One flash purification system (Biotage, Sweden), equipped with a reverse phage preparation column (340 g, LiChroprep C18, particle size 40–63 µm, Merck) and eluted with solvent systems consisting of water: acetonitrile (ACN) (100:1 → 0:100) to yield fourteen fractions according to their TLC (Figure 1). F1 (2 g), F2 (39.8 g), F3 (3.2 g), F4 (1.3 g), F5 (5.4 g), F6 (10.5 g), F7 (12 g), F8 (3.0 g), F9 (3.2 g), F10 (1.2 g), F11 (3.8 g), F12 (1.3 g), F13 (0.1 g), and F14 (0.1 g) were obtained from the APB. Among the fractions, F6, F7, and F10 showed strong NO inhibitory activity in the LPS-treated RAW 264.7 macrophage group, respectively (Table 1). Fraction F6 (10.5 g) was subjected to C18 silica gel column chromatography (100 g) and eluted with water-dichloromethane (100:0 → 0:100) to generate sub-fractions F6-I~F6-XII. Through further purification of sub-fraction F6-IX (7.4 g) and F6-XI (5.1 g) over silica gel, using a gradient solvent system of CHCl_3_-MeOH (20:1 → 0:100), compounds **4** (74.1 mg) and 5 (54.0 mg) were obtained, respectively. Fraction F7 (12.0 g) was subjected to C18 silica gel column chromatography (100 g) and eluted with water-dichloromethane (100:1 → 0:100) using an Isolera One flash purification system to yield fractions F7-I to F7-VII. The highly potent fraction, F7-II, underwent further purification through C18 silica gel column chromatography. It was eluted with a water-dichloromethane gradient (100:1 → 0:100) using an Isolera One flash purification system, leading to the isolation of twelve fractions, F7-II1 to F7-II12. From the active fraction, F7-II4, compounds 1 (23 mg) and 2 (8 mg) were obtained through silica gel CC, using CHCl_3_-MeOH (20:1 → 0:100) as the elution solvent. Fraction F7-II5 (0.7 g), processed through silica gel column chromatography (CC) using CHCl_3_-MeOH (20:1 → 0:100), resulted in the isolation of compound **3** (197 mg) and **4** (15.2 mg), along with compound **5** (13.2 mg). Fraction F7-II11 (0.3 g) underwent silica gel CC (CHCl_3_-MeOH, 20:1 → 0:100), leading to the isolation of compound **6** (8.0 mg). Fraction F10 (1.2 g) was subjected to silica gel column chromatography, eluted with CHCl_3_-MeOH (50:1 → 100:1), to obtain three fractions F10-I~F10III. Further purification of fraction F10-II on Sephadex LH-20 (MeOH) yielded compound **7** (28 mg).

### 3.4. LC-ESI-MS Analysis

LC-MS were carried out on a Shimadzu LCMS-8050 mass spectrometer (Shimadzu, Tokyo, Japan) and on a Waters-Micromass quadrupole-time of flight Ultima TM Global instrument (Waters, Manchester, UK) spectrometer equipped with a DAD detector (set at 280 nm and 360 nm) and MS via an ESI interface in positive and negative ion scan mode. Chromatographic separations for crude extract and isolates were performed using a Shim-pack GIS-ODS column (4.6 × 250 mm, 5.0 μm, YMC, Japan). The mobile phase consisted of 0.5% formic acid in water (solvent A) and methanol (solvent B). The sample was eluted with the following linear gradient: starting from 20% B to 30% B in 5 min, to 50% B in 10 min, to 70% B in 15 min, to 100% B in 20 min, and to 20% B in 35 min. Flow rate was set 0.8 mL/min and the injection volume was set at 10 μL. The column temperature was maintained at 35 °C. The MS parameters were as follows: Q3 scan mode, capillary temperature of 250 °C, sheath gas (N2) flow rate of 3 L/min, aux gas (N2) flow rate of 10 L/min, ion spray voltage of 2.5 kV. Full MS scans in the FT cell were acquired in the range of *m*/*z* 100–975. The isolation window was set at *m*/*z* 1.0, and the stepped collision energy was used at 20, 40, and 60 eV. Xcalibur (Ver. 4.0) was employed for data collection and analysis.

### 3.5. Cell Culture

RAW 264.7 cells (KCLB No. 40071) were used to investigate the anti-inflammatory compounds of *A. pilosa*. RAW 264.7 cells were cultured in Dulbecco’s modified Eagle’s medium (Hyclone, Logan, UT, USA) containing 10% fetal bovine serum (FBS; Gibco, Grand Island, NY, USA) and 1% penicillin streptomycin solution (100 U/mL penicillin and 100 µg/mL streptomycin in 0.85% NaCl) (Invitrogen, Carlsbad, CA, USA) at 37 °C in a saturated atmosphere with 5% CO_2_ and distilled water.

### 3.6. Cell Viability and NO Inhibitory Activity

To evaluate the NO inhibitory activity of samples at non-cytotoxic concentrations, the cell cytotoxicity of compounds **1**–**7** was estimated by MTT assay [28] with some modifications. Briefly, RAW 264.7 cells were seeded in 96-well plates (2 × 10^5^ cells/ well) and stabilized for 24 h. The attached cell was pre-treated with different concentrations (0–40 µM) of sample or DMSO (blank) for 20 h. For MTT assay, MTT solution (5 mg/mL) was added to the well plate and reacted for 4 h at 37 °C, and then cells were lysed with 100 µL DMSO. The MTT formazan in each well was used to measure the absorbance at 550 nm using a UV/Vis spectrophotometer (BioTek, Power Wave XS, Hampton, NH, USA). The level of NO produced in the LPS-treated RAW 264.7 cells treated with the samples was measured according to previously reported method [29] with some modifications. Briefly, RAW 264.7 cells were seeded in 96-well plates (2 × 10^5^ cells/well) and stabilized for 24 h. The cells were pre-treated with or without various concentrations (0–20 µM) of sample for 4 h and then stimulated with 1 µg/mL LPS for 20 h. The amount of NO in the supernatants was determined using the Griess reagent. The same amounts of supernatant and 2% sulfanilamide in distilled water were reacted for 5 min, and then the same amount of 0.2% naphthylethylenediamine dihydrochloride in 4% H_3_PO_4_ was mixed. After 10 min, the absorbance of the formed chromophore was read at 540 nm.

In order to statistical analysis, all measurements were performed in triplicate. The results were subjected to variance analysis using Sigma Plot to analyze statistical significance of the differences. Differences were considered significant at *p* < 0.05.

## 4. Conclusions

Employing the activity-guided isolation methodology, seven potent anti-inflammatory compounds were isolated from the butanol fraction of *Agrimonia pilosa* and identified as quercetin-7-*O*-β-d-rhamnoside (**1**), apigenin-7-*O*-β-d-glucopyranoside (**2**), kaempferol-7-*O*-β-d-glucopyranoside (**3**), quercetin (**4**), kaempferol (**5**), apigenin (**6**), and apigenin-7-*O*-β-d-glucuronide-6″-butylester (**7**). Compound **7** exhibited rare occurrence [30]. The flavones and flavonols isolated in this study represent the first identification of NO inhibitors within this plant [31,32]. Notably, all compounds demonstrated significantly stronger NO inhibitory activity compared to previously reported counterparts [25]. This means that the NO inhibitory activity observed in *A. pilosa* is attributed to the flavones and flavonols isolated in this study. The systematic activity-guided isolation approach proved to be an effective strategy for elucidating the active principle of *A. pilosa.* Compound **7** emerged as a novel anti-inflammatory agent, ascertained through its inaugural demonstration of nitric oxide inhibition. A systematic investigation into the structure-activity relationship (SAR) was undertaken using eight flavonoids present in *A. pilosa*, unveiling the critical influence of the number of hydroxyl groups and glycosylation patterns in flavonoids on nitric oxide (NO) inhibitory activity. These findings propose *A. pilosa* as a promising reservoir of potent anti-inflammatory agents.

## Figures and Tables

**Table 1:** NO inhibitory activities in LPS-treated RAW 264.7 macrophage cells of the fractions from *A. pilosa* L.

Frac.	IC_50_ (μg/mL)	Frac.	IC_50_ (μg/mL)	Frac.	IC_50_ (μg/mL)	Frac.	IC_50_ (μg/mL)
F1	>100	F6-I	77.2 ± 2.9	F7-I	51.3 ± 3.7	F7-II1	73.3 ± 2.9
F2	>100	F6-II	43.3 ± 3.7	F7-II	9.3 ± 1.3	F7-II2	>100
F3	>100	F6-III	34.6 ± 3.1	F7-III	35.9 ± 1.8	F7-II3	30.5 ± 1.2
F4	>100	F6-IV	48.2 ± 0.5	F7-IV	35.8 ± 2.1	F7-II4	6.5 ± 1.4
F5	44.9 ± 0.6	F6-V	46.3 ± 0.6	F7-V	73.7 ± 0.8	F7-II5	3.7 ± 0.6
F6	12.8 ± 1.4	F6-VI	54.5 ± 1.8	F7-VI	48.9 ± 9.6	F7-II6	73.8 ± 2.4
F7	9.3 ± 0.7	F6-VII	58.9 ± 4.6	F7-VII	63.6 ± 9.6	F7-II7	63.6 ± 0.4
F8	44.5 ± 2.0	F6-VIII	25.4 ± 4.8			F7-II8	61.8 ± 1.7
F9	59.1 ± 2.1	F6-IX	18.6 ± 2.7			F7-II9	41.0 ± 3.3
F10	8.5 ± 1.0	F6-X	36.9 ± 1.9			F7-II10	32.6 ± 2.4
F11	45.6 ± 4.0	F6-XI	7.7 ± 1.8			F7-II11	1.4 ± 0.7
F12	>100	F6-XII	47.8 ± 2.5			F7-II12	37.4 ± 2.0
F13	57.0 ± 2.1					F7-II13	48.3 ± 2.9
F14	>100					F7-II14	32.2 ± 4.6
						F7-II15	36.8 ± 2.8

**Table 3:** Comparison of NO inhibitory effects on LPS-treated RAW 264.7 cells of compounds (**1**–**7**) from *A. polisa*.

No.	Compound	NO Inhibitory Activity (IC_50_, μM)
1	Quercetin-7-*O*-β-d-rhamnoside	31.26 ± 0.06
2	Apigenin-7-*O*-β-d-glucopyranoside	8.03 ± 0.26
3	Kaempferol-7-*O*-*β*-d-glucopyranoside	22.24 ± 0.15
4	Quercetin	19.51 ± 0.13
5	Kaempferol	15.75 ± 0.39
6	Apigenin	1.43 ± 0.59
7	Apigenin-7-*O*-β-d-glucuronide-6″ -butylester	14.68 ± 0.09

**Table 4:** The structure-activity relationship of flavonoids from *A. pilosa*.

Aglycone	NO Inhibitory Activity (IC50, µM)	Glycone (Glc)	NO Inhibitory Activity (IC50, µM)
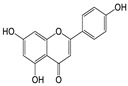 Apigenin	3.69 ± 0.34	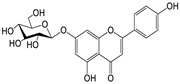 Apigenin-7-*O*-Glc	8.03 ± 1.26
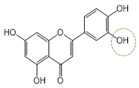 Luteolin	4.62 ± 0.43	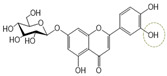 Luteolin-7-*O*-Glc	14.37 ± 1.84
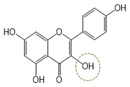 Kaempferol	14.43 ± 0.23	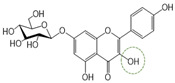 Kaempferol-7-*O*-Glc	22.24 ± 2.14
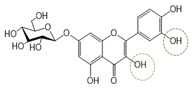 Quercetin	19.50 ± 1.71	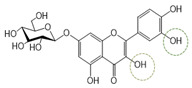 Quercetin-7-*O*-Glc	31.27 ± 3.75

## Data Availability

The data presented in this study are available in article and Supplementary Materials.

## Supplementary Materials

The following supporting information can be downloaded at: https://www.mdpi.com/article/10.3390/molecules29020283/s1, Figure S1: HPLC chromatogram of compound **7** (MeOH) at 360 nm (Purity 91%); Figures S2–S5: 1D, 2D NMR spectra of compound **7** (500 MHz, MeOH-d4); Figure S6: HR-ESI-MS spectra of compound **7**. Table S1: ^1^H NMR Spectroscopic Data for Compounds (**1**–**6**).
