# Supplementary material for: Anti-Inflammatory Flavonoids from Agrimonia pilosa Ledeb: Focusing on Activity-Guided Isolation
Source: Molecules. 2024 Jan 5;29(2):283. doi: 10.3390/molecules29020283 (PMC10819444; doi:10.3390/molecules29020283)
Supplement: Supplementary file 1 [file molecules-29-00283-s001.zip › molecules-2732224-supplementary.pdf]

Supporting Information

Compound 7: Apigenin-7-O-β-D-glucuronide-butyl-ester

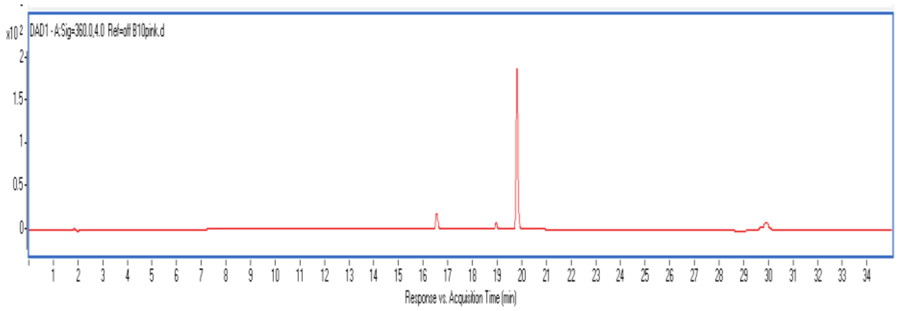

Figure S1. HPLC chromatogram of compound **7** (MeOH) at 360 nm (Purity 91 %).

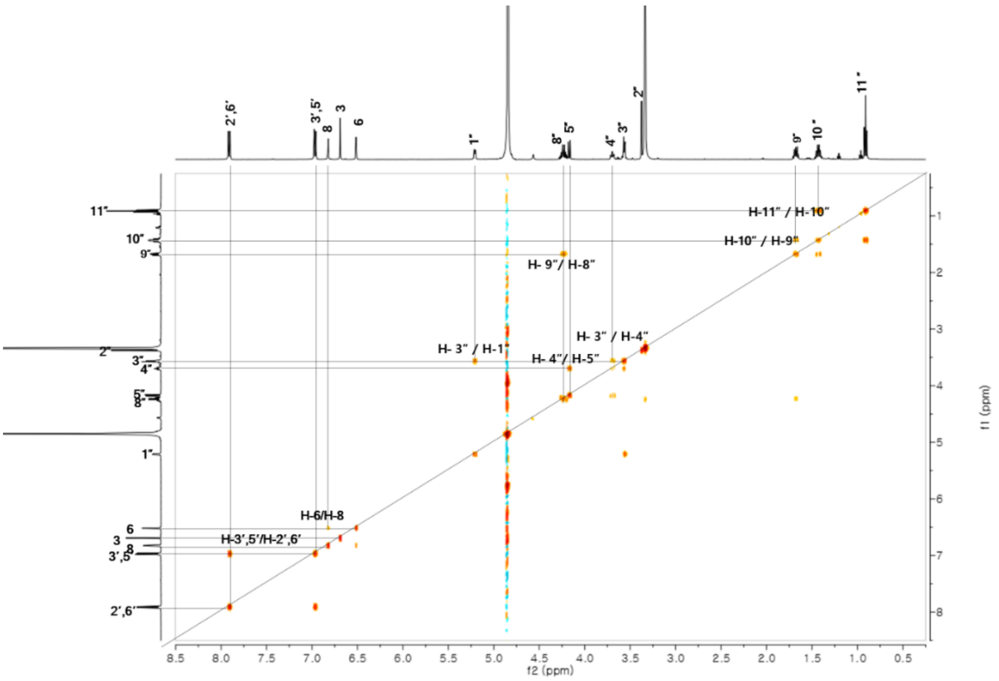

Figure S2. ^{1}H-^{1}H COSY spectrum of compound **7** (MeOH-d_4).

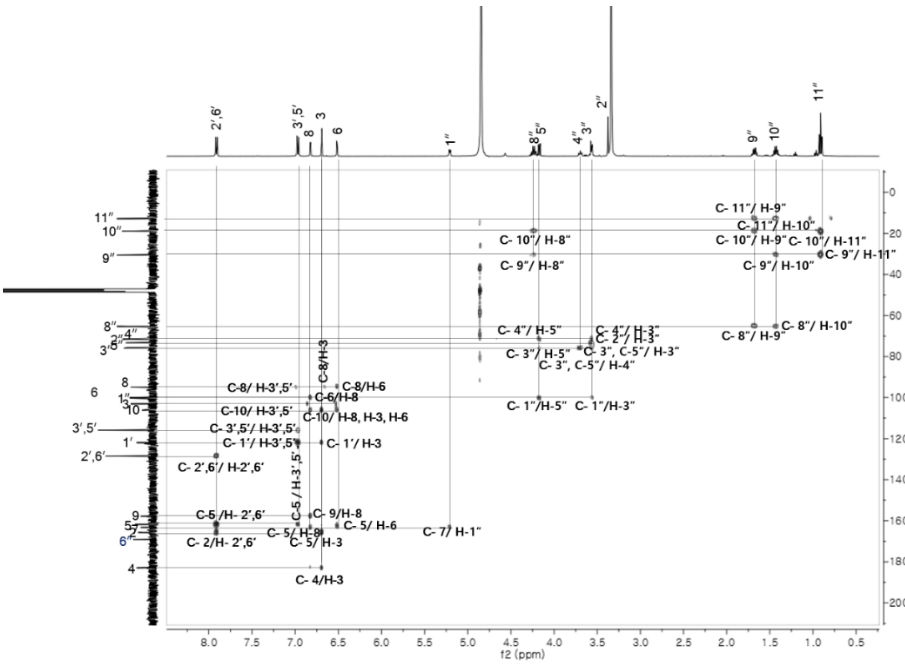

Figure S3. HMBC spectrum of compound **7** (MeOH-d_4).

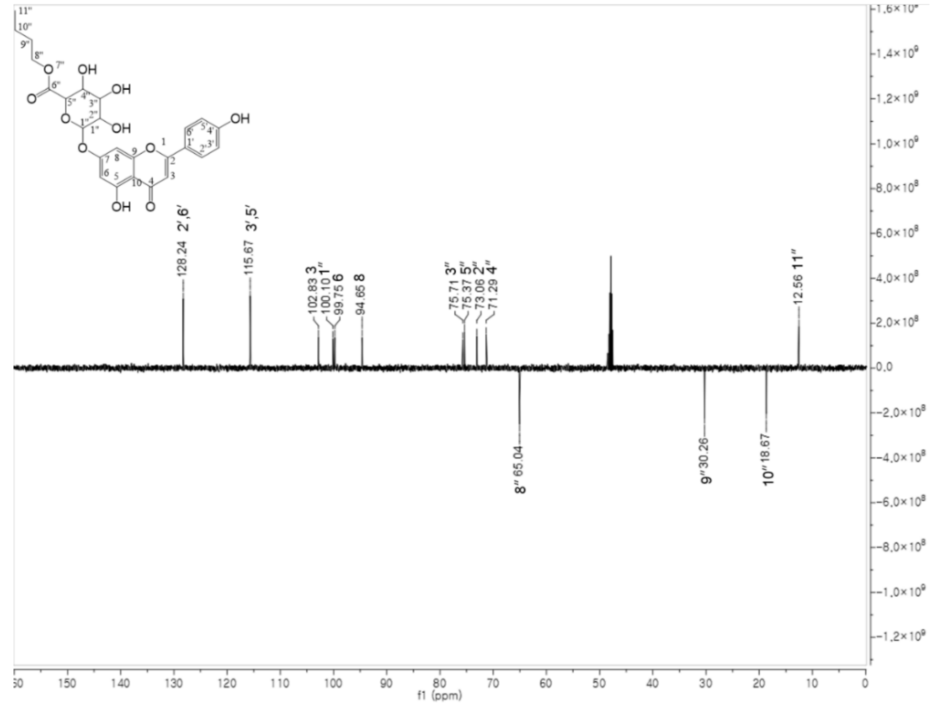

Figure S4. DEPT spectrum of compound **7** (MeOH-d_4).

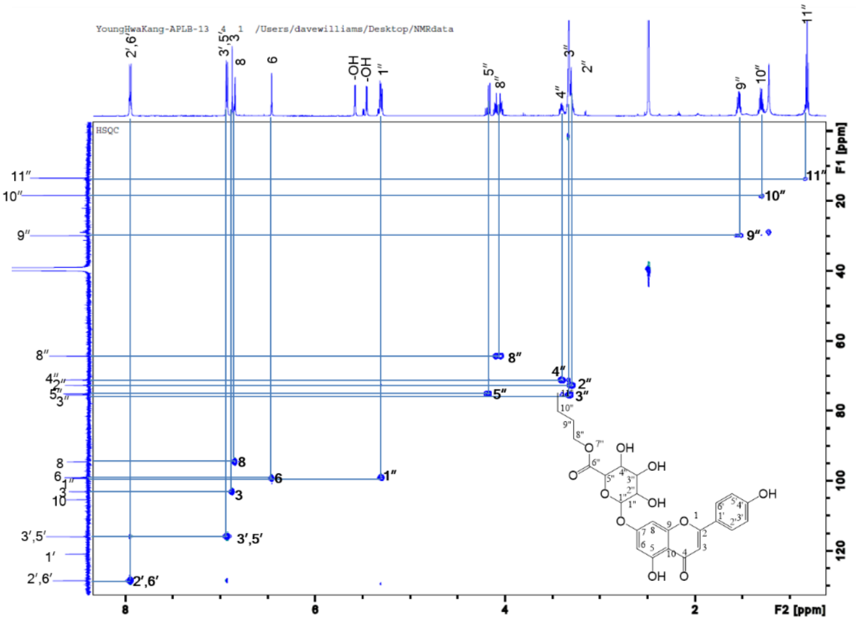

Figure S5. HSQC spectrum of compound **7** (MeOH-d_4).

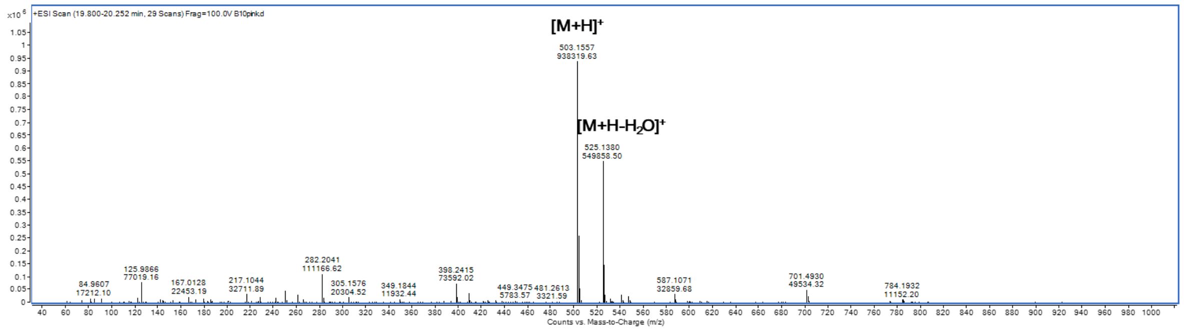

Figure S6. HR-ESI-MS spectra of compound **7**.

Table S1. ^{1}H NMR Spectroscopic Data for Compounds (**1-6**) [a].

Compound	Quercetin-7-*O*-*β*-D-rhamnoside (**1**)	Apigenin-7-*O*-*β*-D-glucopyranoside (**2**)	Kaempferol-7-*O*-*β*-D-glucopyranoside (**3**)
Position	δ_H (mult, *J*, int)[b]	δ_H (mult, *J*, int)[c]	δ_H (mult, *J*, int)[c]
Aglycone			
6	6.19 (d, *J* = 2.0 Hz, 1H)	6.46 (d, *J* = 2.2 Hz, 1H)	6.44 (d, *J* = 2.0 Hz, 1H)
8	6.35 (d, *J* = 1.9 Hz, 1H)	6.85 (d, *J* = 2.2 Hz, 1H)	6.83 (d, *J* = 2.0 Hz, 1H)
2′	7.37 (d, *J* = 2.0 Hz, 1H)	7.96 (d, *J* = 8.9 Hz, 2H)	7.94 (d, *J* = 8.7 Hz, 2H)
3′		6.95 (d, *J* = 8.9 Hz, 2H)	6.93 (d, *J* = 8.7 Hz, 2H)
5′	6.95 (d, *J* = 8.3 Hz, 1H)	6.95 (d, *J* = 8.9 Hz, 2H)	6.93 (d, *J* = 8.7 Hz, 2H)
6′	7.33 (dd, *J* = 2.0 Hz, 1H)	7.96 (d, *J* = 8.9 Hz, 2H)	7.94 (d, *J* = 8.7 Hz, 2H)
Glucopyranoside			
1″	5.35 (d, *J* = 7.3 Hz, 1H)	5.07 (d, *J* = 7.5 Hz, 1H)	5.06 (d, *J* = 7.3 Hz, 1H)
2″	3.38 (t, *J* = 4.7 Hz, 1H)		
3″	4.22 (d, *J* = 9.4 Hz, 1H)	3.33-3.20 (m, 3H)	3.25-3.16 (m, 3H)
4″	3.44 (dd, *J* = 9.4, 6.1 Hz, 1H)		
5″	4.22 (d, *J* = 9.4 Hz, 1H)	3.71 (d, *J* = 9.9 Hz, 1H)	3.62 (d, *J* = 9.4 Hz, 1H)
6″	3.36-3.31 (m, 2H)	3.20 (m, 1H)	3.16 (m, 1H)
$-CH_3$	0.97 (d, *J* = 6.1 Hz, 3H)		

[a] Recorded in MeOH-$d_4$and DMSO-d_6 at 500/125 MHz (TMS as internal standard); chemical shifts, multiplicity, and coupling constants (*J*, Hz) were assigned by means of ^{1}H [b] MeOH-d_4, [c] DMSO-d_6

Table S1. (Continued). ^{1}H NMR Spectroscopic data for Compounds (**1-6**)[a].

Compound	Quercetin (**4**)	Kaempferol (**5**)	Apigenin (**6**)
Position	δ_H (mult, *J*, int)[b]	δ_H (mult, *J*, int)[c]	δ_H (mult, *J*, int)[c]
Aglycone			
3			6.77 (s, 1H)
6	6.20 (d, *J* = 2.0 Hz, 1H)	6.48 (d, *J* = 2.0 Hz, 1H)	6.22 (d, *J* = 2.2 Hz, 1H)
8	6.43 (d, *J* = 2.0 Hz, 1H)	6.86 (d, *J* = 2.0 Hz, 1H)	6.51 (d, *J* = 2.2 Hz, 1H)
2′	7.67 (d, *J* = 2.0 Hz, 1H)	7.95 (d, *J* = 8.6 Hz, 2H)	7.93 (d, *J* = 8.9 Hz, 2H)
3′		6.94 (d, *J* = 8.8 Hz, 2H)	6.94 (d, *J* = 8.9 Hz, 2H)
5′	6.90 (d, *J* = 8.5 Hz, 1H)	6.94 (d, *J* = 8.8 Hz, 2H)	6.94 (d, *J* = 8.9 Hz, 2H)
6′	7.54 (dd, *J* = 8.5, 2.0 Hz, 1H)	7.95 (d, *J* = 8.6 Hz, 2H)	7.93 (d, *J* = 8.9 Hz, 2H)

[a] Recorded in MeOH-$d_4$and DMSO-d_6 at 500/125 MHz (TMS as internal standard); chemical shifts, multiplicity, and coupling constants (*J*, Hz) were assigned by means of ^{1}H [b]MeOH-d_4, [c]DMSO-d_6
